# Inkjet Printing of Electrodes on Electrospun Micro- and Nanofiber Hydrophobic Membranes for Flexible and Smart Textile Applications

**DOI:** 10.3390/polym14225043

**Published:** 2022-11-21

**Authors:** Zuzanna J. Krysiak, Hamed Abdolmaleki, Shweta Agarwala, Urszula Stachewicz

**Affiliations:** 1 Faculty of Metals Engineering and Industrial Computer Science, AGH University of Science and Technology, 30-059 Krakow, Poland; 2 Department of Electrical and Computer Engineering, Aarhus University, 8000 Aarhus, Denmark

**Keywords:** printed electronics, inkjet printing, electrospinning, fibers, hydrophobicity, cells, membrane

## Abstract

With the increasing demand for smart textile and sensor applications, the interest in printed electronics is rising. In this study, we explore the applicability of electrospun membranes, characterized by high porosity and hydrophobicity, as potential substrates for printed electronics. The two most common inks, silver and carbon, were used in inkjet printing to create a conductive paths on electrospun membranes. As substrates, we selected hydrophobic polymers, such as polyimide (PI), low- and high-molecular-weight poly (vinyl butyral-co-vinyl alcohol-co-vinyl acetate) (PVB) and polystyrene (PS). Electrospinning of PI and PVB resulted in nanofibers in the range of 300–500 nm and PVB and PS microfibers (1–5 μm). The printed patterns were investigated with a scanning electron microscope (SEM) and resistance measurements. To verify the biocompatibility of printed electrodes on the membranes, an indirect cytotoxicity test with cells (MG-63) was performed. In this research, we demonstrated good printability of silver and carbon inks on flexible PI, PVB and PS electrospun membranes, leading to electrodes with excellent conductivity. The cytotoxicity study indicated the possibility of using manufactured printed electronics for various sensors and also as topical wearable devices.

## 1. Introduction

Direct-write technologies have disrupted the manufacturing of electronic devices over the last decade [1]. This simple and affordable strategy uses direct patterning of conducting and functional inks on a wide variety of substrates [2]. Inkjet [3], electrohydrodynamic [4] and aerosol jet [5] printing are common droplet-based printing techniques that fall under additive manufacturing technology [6]. Various devices such as transistors, batteries, solar cells, sensors and health monitoring have been fabricated using these techniques [7,8,9,10]. Inkjet technology is a digital, noncontact direct-write technique under printed electronics that has shown great promise to fabricate flexible, bent and stretchable electronics [11]. Printing in the drop-on-demand (DOD) mode accelerates the development of new fields of applications in smart textiles [12]. In inkjet printing, the drop ejection is controlled by trigger signals passing through actuators in the printhead. The actuators used in DOD inkjet printing are either thermal or piezoelectric. Thermal actuators heat up upon the passage of the trigger signal, which leads to fluid expansion followed by drop ejection; while in piezoelectric actuators, the trigger signal causes electromechanical displacement in the piezoelement to thrust out an ink droplet [13,14]. Printed patterns on a substrate need to undergo postprocessing steps such as drying and sintering to, respectively, remove solvents and binders from functional inks and generate conductive paths [15].

Printing quality depends on various process parameters, such as droplet size, nozzle diameter, the distance between the nozzle and substrate, ink viscosity and surface tension [12,16]. Substrates can be selected from both synthetic and natural materials having flexibility, heat resistance, surface smoothness, adjustable thickness and low cost [17]. Although the inkjet technique is becoming more and more popular, there are still some limitations [18], and selecting the best substrates is still being investigated [19,20,21]. Moreover, choosing an appropriate sintering method for a particular substrate is demanding, as the postprocessing methods should not damage the substrate [13,22,23]. Electrospun fibers have been explored as printing screen stencils [24], but using them as the substrate for printed electronics due to high surface roughness and porosity is still challenging. However, fibrous membranes can have excellent mechanical properties [25,26] and high flexibility [27,28], which are advantageous for wearable sensors [29,30,31,32,33]. With electrospinning, it is possible to produce meshes with a high surface-area-to-volume ratio, great permeability and adjustable functionality [31,34]. Recent advancement in the large-scale production of electrospun fibers [35,36,37,38] has led to extensive research for the deployment of these materials for different applications [39,40,41,42,43]. Electrospinning provides the production of polymeric fibers with a broad range of diameters, from nano- to microscale, affecting the pore size and porosity of the membranes [44,45,46]. Moreover, the wetting properties of the electrospun mats can be controlled by selecting hydrophilic or hydrophobic polymers [47,48,49]. The surface properties of electrospun polymer fibers can be controlled via electrospinning parameters or by further modifications [50]. For instance, inkjet printing was applied to deposit silver nanoparticles (AgNPs) on polyurethane (PU) fibers to manufacture an antimicrobial membrane for water purification [51]. Poly(caprolactone)-poly(glycerol sebacate) (PCL-PGS) electrospun fibers were printed with silver ink to create stretchable and biodegradable electronics. Additionally, the manufactured conductive materials did not show cytotoxicity on NIH 3T3 fibroblasts, indicating their potential application as smart dermal patches [52].

This study aims to perform a visibility study to explore the suitability of commonly used electrospun fibers as substrates for inkjet printing. We selected polyimide (PI), poly(vinyl butyral-co-vinyl alcohol-co-vinyl acetate) (PVB) nanofibrous and PVB and polystyrene (PS) microfibrous membranes as substrates for inkjet printing with silver and carbon ink. Fiber diameter is a crucial parameter in electrospun membranes, as it determines the pore size and surface roughness. We studied the influence of fiber diameter on the quality of inkjet-printed electrodes with both silver and carbon ink. The detailed morphology and electrical resistance of printed layers were analyzed to justify the printing quality. An indirect cytotoxicity assay was also performed to investigate the biocompatibility of printed electrodes. We demonstrated the importance of polymer and ink selection, especially concerning their required sintering temperature and particle size. Most importantly, the geometry of membranes related to the fiber diameter and pore size defines the ink penetration and further application of obtained printed electrodes on porous substrates. We believe this work can open up new routes for deploying electrospun fibers as flexible substrates for printed electronics with a huge potential in smart textiles.

## 2. Materials and Methods

### 2.1. Electrospinning

Poly(vinyl butyral-co-vinyl alcohol-co-vinyl acetate) (PVB) and polystyrene (PS) were obtained from Sigma-Aldrich (St. Louis, MO, USA); polyimide (PI) was provided by Ensinger Sintimid GmbH (Graz, Austria). Prior to the solution preparation, PS and PI were dried at T = 30 °C for 3 h and at T = 50 °C for 4 h, respectively (Drying Oven, POL-ECO Aparatura, Wodzisław Śląski, Poland). Low- (LM_w_, M_w_ = 70,000–100,000 g·mol^−1^) and high- (HM_w_, Mw = 170,000–250,000 g·mol^−1^) molecular-weight PVB was dissolved up to 10 wt% in methanol, *N*,*N*-dimethylformamide (DMF) and dimethyl sulfoxide (DMSO) mixed in ratio 5:4:1 for LM_w_ and ratio 4:5:1 for HM_w_ and stirred for 3 h at 1000 rpm in 35 °C. Fibers obtained from mentioned solutions were referred to as nano- and microfibers. The 25 wt% solution of PS (350,000 g·mol^−1^) in DMF was prepared and 12 wt% PI in DMSO and dimethylacetamide (DMAc) mixed in ratio 7:3. Solutions were stirred for 4 h at 500 rpm in 20 °C and 12 h at 250 rpm in 35 °C, respectively.

**Figure 1:**
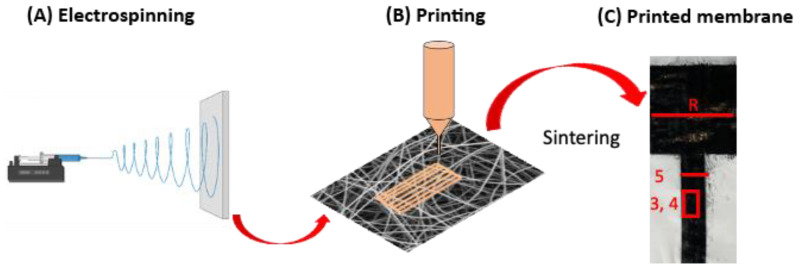
The schematic diagram representing all stages of fabrication of conducting path on electrospun fibers, consisting of (**A**) electrospinning the polymer membrane, (**B**) printing conductive inks directly on electrospun membranes and (**C**) sintering processes of printed ink on polymer fibers with marked locations of image regions via SEM in Figure 3, Figure 4 and Figure 5 on the printed profiles for all samples.

Fibers were manufactured via electrospinning (Figure 1A) with climate control (TechNOVA, Beijing, China) and deposited for 1.5 h on the rotating (5 rpm) collector covered with baking paper. Electrospinning parameters for all samples are presented in Table 1.

### 2.2. Inkjet Printing and Sintering

A Dimatix DOD inkjet printer DMP-2850 (FujiFilm, Santa Clara, CA, USA), equipped with a drop-watcher and a fiducial camera, was used to print water-based silver or carbon nanoparticles inks (JS-A101A, JR-700LV, NovaCentrix, Austin, TX, USA). The silver ink (η = 5–7 mPa·s, σ = 19–30 mN·m^−1^) was passed through PP filter with glass fibers prefilter (pore size 0.2 µm, Pall Life Sciences, USA) before printing and then injected into an inkjet cartridge with drop volume of 1 pL (Dimatix Model Fluid, FujiFilm, Santa Clara, CA, USA). For carbon-based ink (η = 3–6 mPa·s, σ = 30–35 mN·m^−1^), 10 pL inkjet cartridges were used. Nano and micro PVB, PS and PI fibers were used as a substrate for printing, see Figure 1. Prior to printing, both cartridge and platen were heated up to 40 °C and substrate was tightly fixed to the platen with the tape. The applied trigger voltage for silver and carbon inks was 32 and 40 V, respectively. The distance between the platen and cartridge was set to 700 μm for PVB membranes with silver ink and 1000 μm for carbon ink. For PS and PI membranes, the cartridge–platen distance was 1200 μm and 1000 μm, respectively, for both inks. For all membranes, two layers of ink were printed as summarized in Table 2.

To obtain homogenous and conductive layers on the substrates by removing unnecessary additives, sintering was performed. Nano- and microfiber-based PVB membranes were sintered for 15 h at 70 °C in the furnace (Nabertherm GmbH, Lilienthal, Germany). PS and PI membranes were sintered on hot plate (IKA C-MAG HS7, Staufen, Germany) for 20 min at 90 °C and 10 min at 140 °C for silver ink, respectively. In the case of carbon ink, PS membrane was sintered for 1 h at 90 °C, while PI for 15 min at 200 °C.

### 2.3. Characterization of Printed Layers on Electrospun Membranes

Membrane morphology and printing quality were investigated using scanning electron microscope (SEM, Merlin Gemini II, Zeiss, Munich, Germany) with an accelerating voltage of 3 kV and 120 pA current at a working distance of 5–8 mm. Prior to imaging, the samples were sputtered with 8 nm Au layer (Q150RS, Quorum Technologies, Laughton, UK). Additionally, the samples were immersed in the liquid N_2_ to obtain the cross-section by cutting the frozen sample with a scalpel. The average thickness of ink penetration into the electrospun membranes was measured from obtained cross-section images using ImageJ software (ver. 1.53v, National Institutes of Health, Bethesda, MD, USA). The SEM investigation was performed as marked in Figure 1C; the cross-section images were taken from region 5.

The wetting properties of electrospun membranes were determined. The contact angle was analyzed with deionized water (DI water, Spring 5UV purification system Hydrolab, Straszyn, Poland). Images were taken with a Canon EOS 700D camera with EF-S 60 mm f/2.8 Macro USM zoom lens within 3 s after droplet (3 μL) deposition on the membrane. The contact angle was analyzed using an MB-Ruler (ver. 5.3, Iffezheim, Germany), and the mean value was calculated from 10 measurements.

The resistance of all sintered samples was measured with two-probe digital multimeter (Keithley, Beaverton, OR, USA). The measurements were performed for all samples with the same distance between the electrodes (1.5 cm), see marked line with R in Figure 1C. The average resistance and errors based on standard deviation were calculated from 3 separate measurements at different locations for each sample type.

### 2.4. Indirect Cytotoxicity Test

The samples with a diameter of 15 mm were cut from pristine and printed PI and PVB nanofibers, PVB and PS microfibers. Next, they were sterilized with UV light for 30 min and incubated in 2 mL of complete cell culture medium composed of Dulbecco’s modified Eagle medium (DMEM with 4.5 g/L D-glucose, Biological Industries, Kibbutz Beit-Haemek, Israel), supplemented with 10% of fetal bovine serum (FBS, Biological Industries, Israel), 2% of antibiotics (penicillin-streptomycin, Biological Industries, Kibbutz Beit-Haemek, Israel), 1% of aminoacids (Mem nonessential amino acid solution 100×, Sigma-Aldrich, St. Louis, MO, USA) and 1% of L-glutamine solution (Biological Industries, Kibbutz Beit-Haemek, Israel) for 72 h at 37 °C, RH = 90% and 5% of CO_2_ (Memmert GmbH + Co.KG, INC 108med, Schwabach, Germany). Afterward, samples were collected and stored at 4 °C. Human osteoblasts cells (MG-63) were seeded on 96-well plate with density of 4 × 10^3^ per well and cultured for 24 h. Next, cell culture medium was discarded, and osteoblasts were incubated for 24 h with collected supernatants. For positive control, cells were incubated with cell culture medium. For all the solutions, 4 repetitions were performed. After 24 h, 20 μL of CellTiter Blue (Promega, Madison, WI, USA) reagent was added and incubated for 4 h at 37 °C, RH = 90% and 5% of CO_2_. Then, 100 μL of each reaction solution was transferred to the 96-well plate, and the fluorescence was measured (excitation 560 nm/emission 590 nm) (GloMax Discover, Promega, Madison, WI, USA). Analysis of variance (one-way ANOVA followed by Tukey’s posthoc test) was used to determine the level of significance between the samples; the statistical significance was evaluated at *p* < 0.05.

## 3. Results and Discussion

### 3.1. Printability

**Figure 2:**
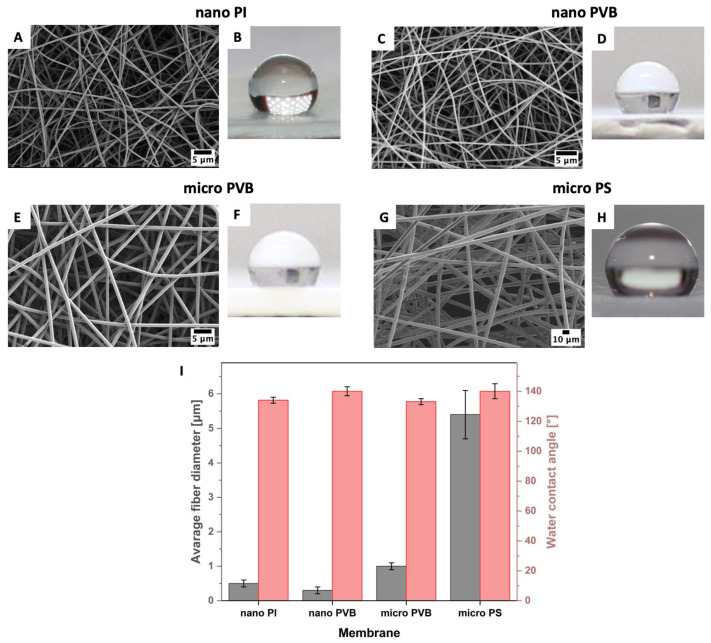
SEM micrographs and water droplet images on electrospun fibers: (**A**,**B**) nano PI, (**C**,**D**) nano PVB, (**E**,**F**) micro PVB (**G**,**H**) and micro PS. (**I**) The column chart indicates the average fiber diameter and water contact angle of nano PI, nano PVB, micro PVB and PS fibers.

The printability of silver and carbon inks was verified on hydrophobic electrospun membranes with a wide range of fiber diameters from 300 nm to 5 µm (Figure 2). According to our previous studies [53,54], the average fiber diameter for PI and PVB nanofibers was 500 ± 70 nm and 335 ± 86 nm, respectively. PVB microfibers were characterized by a higher fiber diameter of 966 ± 92 nm [53], and the PS fibers even reached an average fiber diameter of 5.41 ± 0.29 μm [55].

**Figure 3:**
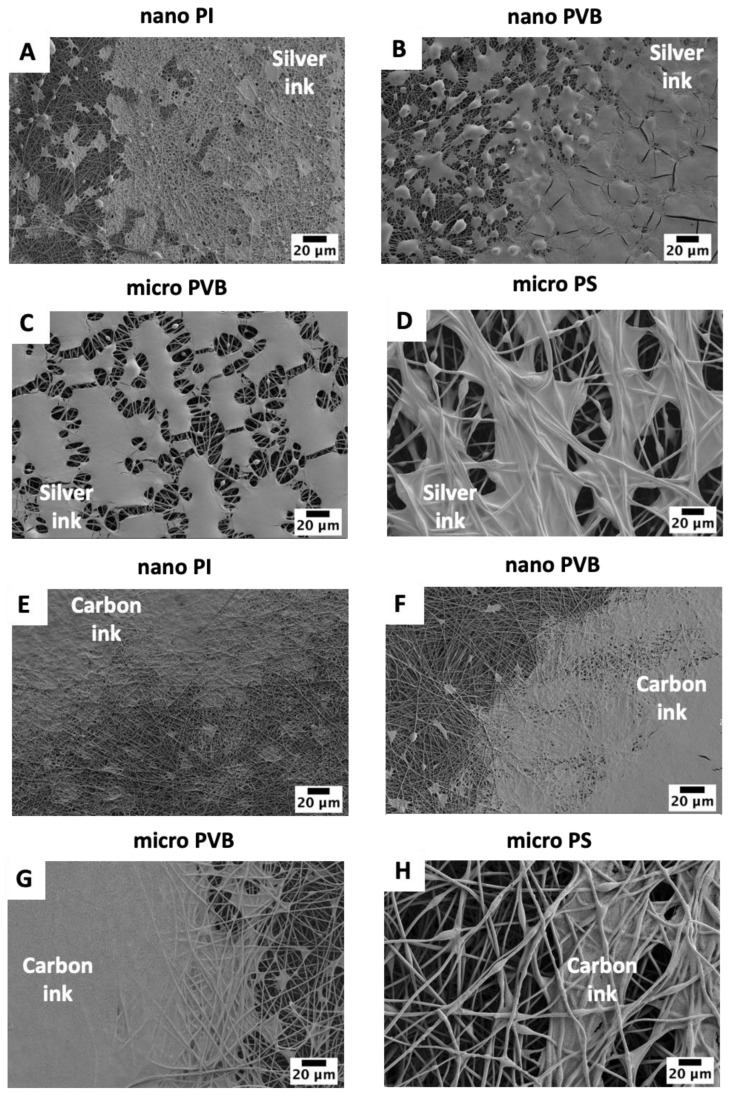
SEM micrographs of electrospun membranes: (**A**,**E**) nano PI and (**B**,**F**) nano PVB, (**C**,**G**) micro PVB and (**D**,**H**) micro PS printed with two layers of silver and carbon ink, respectively. All pictures were taken after sample sintering.

A high-resolution pattern was inkjet printed on the fibrous membranes. The excellent printability of silver inks on nano PI and PVB fibers (Figure 3A,B) can be attributed to the smaller pore diameter and lower surface roughness of these membranes [56]. Silver ink was homogenously distributed and created smooth coverage on the membranes. For PVB and PS microfibers, the greater pore size led to ink penetration in the substrates, thus lower printing quality. The surface tension of silver and carbon ink is much lower than for water to measure the wetting properties [57,58]; therefore, ink droplets on electrospun membranes were spread while compared to hydrophobic contact angles, presented in Figure 1 [53,54,59]. Additionally, the micro PS fibers showed slightly different wetting behavior with ink than the other substrates, as the polymer’s surface free energy varies too. Among all substrates, the surface free energy of PS is the lowest, reaching 25 mJ·m^−2^ [47]. The surface free energy of electrospun fibers can vary for polymer films and electrospun fibers [50], which can affect the drying of inks after printing [21,60]. Small ink droplets with a volume of 1 pL were unable to fully cover the PVB microfiber-based membranes for two printed passes of silver ink. The pores related to the distance between the microfibers in the electrospun mesh are greater than between nanofibers [56]; therefore, printing with silver ink was insufficient in creating a homogenous layer on the membrane surface (Figure 3C). Interestingly, the PS microfiber electrospun mesh based on the microfibers was covered with sliver ink islands, as the ink was entrapped between the fibers and not well-distributed on the PS membrane. The droplets of silver ink have similar behavior to water droplets on the single fiber [61]. Further, as the distance between the fibers is large, the ink penetrates the membrane (Figure 3D).

For carbon ink, detailed observation with SEM micrographs demonstrated differences in the wetting property of fibers and the uniformity of printed electrodes. The two layers of carbon ink printed on nano and micro PVB and nano PI fibers homogeneously covered the electrospun membrane surface (Figure 3E–G). The carbon layer on the micro-PS membrane showed similar morphology as the silver ink. Generally, the droplets of ink were entrapped between the fibers in pores; however, we also observed the wetting effects on the individual single fibers (Figure 3H), indicating that the surface free energy of the electrospun polymer fibers can affect their wettability. In printing, the nozzle diameter directly affects the droplet size, which in turn affects the printing resolution. The small droplets (1 pL) of silver ink were not able to cover the fiber’s surface even when two layers were printed, see Figure 3A–D. In contrast, the droplets of carbon ink were larger (10 pL), creating a uniform coating layer on the electrospun membranes, see Figure 3C–H. The viscosity and surface tension of both inks were similar; the only difference was the needle size during the printing, regulating the size of ink droplets, so the amount of deposited ink too.

**Figure 4:**
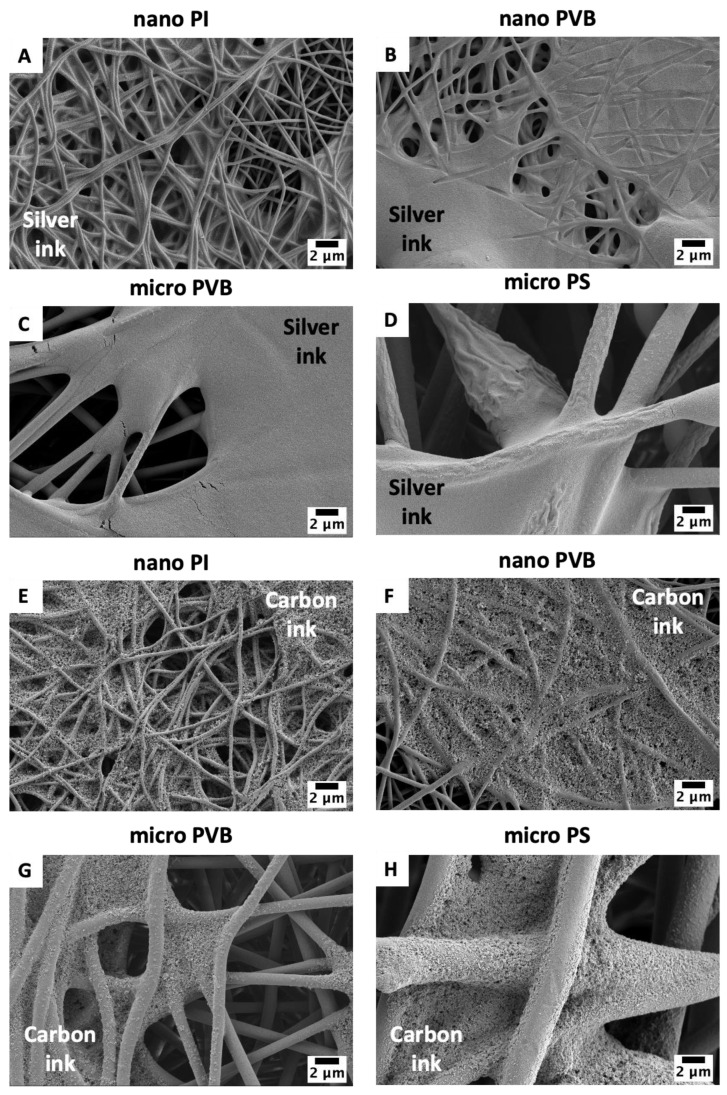
SEM micrographs of electrospun membranes: (**A**,**E**) nano PI and (**B**,**F**) nano PVB, (**C**,**G**) micro PVB and (**D**,**H**) micro PS printed with two layers of silver and carbon ink, respectively. All pictures were taken after sample sintering.

The detailed study of ink and fiber interaction indicated the difference in the morphology of sintered ink on the electrospun membranes. The layer of silver ink was smooth (Figure 4A–D) while carbon ink created a rugged structure (Figure 4E–H). After sintering, both inks were well integrated with the fibers, and for the nano and micro PVB and nano PI fibers, inks were entrapped between the fibers. Macro PS fibers were coated with ink too. Most importantly, the sintering process at the adjusted temperature for all polymers did not affect the morphology of the fibers.

**Figure 5:**
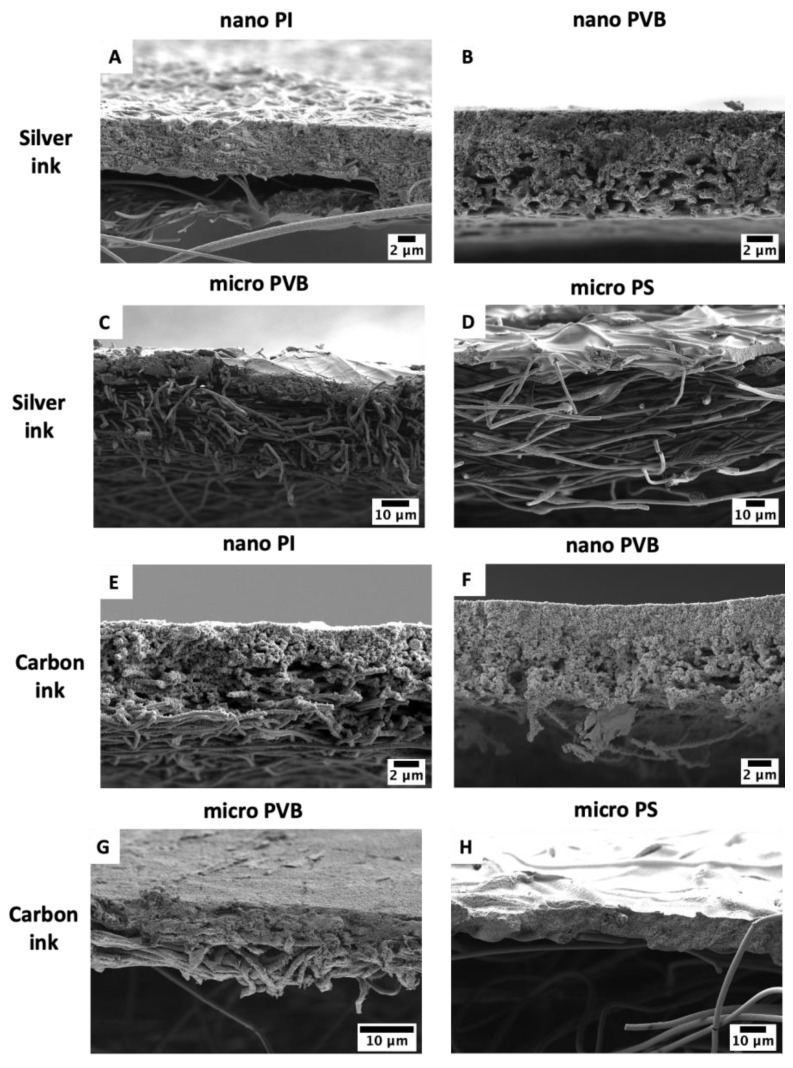
SEM micrographs of cross-section of sintered samples of electrospun membranes based on: (**A**,**C**) nano PI and (**B**,**D**) nano PVB, (**E**,**G**) micro PVB and (**F**,**H**) micro PS printed with two layers of silver and carbon ink, respectively.

To investigate the penetration depth of the inks in the electrospun fibers, a cross-section of each sample was analyzed with SEM. The cross-section micrographs clearly showed the interaction between the fibers and ink from a different perspective. The typical top view of printed layers on nano PI and nano PVB showed limited ink penetration into the membranes (Figure 3). However, cross-sectional images presented in Figure 5A,B,E,F indicated ink infiltration through the electrospun membrane. Ink droplets were able to penetrate the membrane, reaching the bottom of the sample. Additionally, for the microfibers, PVB and PS, cross-section images demonstrated that both inks partially penetrated the membrane, as for nanofibers. The greater the fibers’ diameter, the smaller ink droplets were integrated into the fiber rather than entrapped between the fibers (Figure 5C,D,G,H), which is related to the already discussed wetting properties of electrospun fibers [62]. In the case of the PS membrane, both silver and carbon ink infiltrated close to the sample surface at 7.3 ± 1.3 µm and 10.6 ± 1.0 µm, respectively, see Figure 5D,H. Interestingly, both inks penetrated most of the membranes at similar depths around 7 µm. Except for silver ink in PI nanofibers, which infiltrated up to the 5.6 ± 0.3 µm and carbon ink in PS microfibers up to the 10.6 ± 1.0 µm.

**Figure 6:**
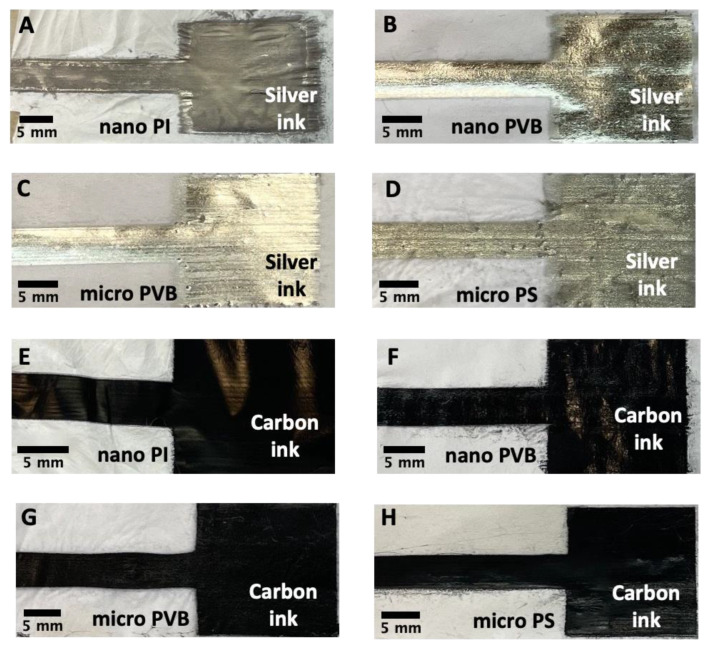
Macroscopic images of electrospun membranes built of nanofibers (**A**,**E**) PI and (**B**,**F**) PVB; microfibers (**C**,**G**) PVB and (**D**,**H**) PS printed with two layers of silver ad carob ink respectively. All pictures were taken after sintering step.

After sintering, a uniform and glossy silver layer was created on the nano and micro PVB fibers (Figure 6B,C); however, printed electrodes on the PI and PS fibers were dim and less uniform (Figure 2A,D). Macroscopic images of electrospun membranes printed with carbon ink did not show significant differences in the printing quality comparing the substrates (Figure 3A,B,E,F). All the printed surfaces were glossy after sintering. Despite the hydrophobic character of electrospun membranes (Figure 2), they demonstrated the absorption capacity of water-based inks. Therefore, both inks presented even distribution and spreading, creating continuous patterns on all electrospun substrates.

### 3.2. Resistance

The glass transition (T_g_) temperature of PVB, PS and PI enables the sintering of silver ink at a temperature close to the optimal sintering temperature (140 °C) stated in the ink datasheet [57]. Therefore, electrospun membranes printed with silver ink can present lower resistance than those with carbon ink. Moreover, silver ink has intrinsically higher conductivity than carbon ink according to the technical data sheet [58]. Cartridges with smaller nozzle diameters were used for printing with silver ink, thus the 1 pL droplets were able to create a more homogenous conductive layer and closely packed conductive structure than carbon ink.

**Figure 7:**
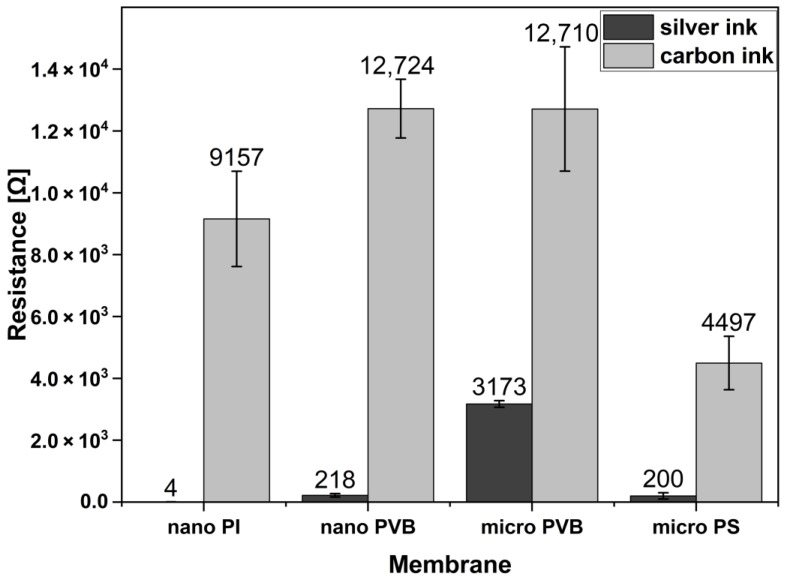
Resistance of silver and carbon conductive path printed on nano PI and nano PVB, micro PVB and micro PS electrospun membranes. On top of the columns, the exact values of the resistance are provided.

The size of silver particles in the ink was in the range of 30–50 nm [57] while carbon was in the range of 120–150 nm [58]. Therefore, a bigger printhead was used for carbon ink to pass through the nozzles in the cartridge. These settings were limited by the commercial printing setup used for our studies, but it affected the quality of printing that has to be taken into account when electrospun membranes are used as substrates for printing [63]. Moreover, small error bars also confirmed the uniformity of the silver printed layer, see Figure 7. The sintering temperature of carbon ink (250 °C) is significantly higher than for silver and destructive for the used polymers. Hence, the maximum applied temperature was set at 200 °C; consequently, electrospun meshes printed with carbon ink were less conductive than those with silver. Furthermore, the homogeneity of printing was also reduced, which is visible by the high error bars, see Figure 7. The wetting of the deposited ink droplets on the substrate was influenced by the surface roughness and hydrophobicity, which play a crucial role in printing quality [7,64]. The roughness of electrospun membranes is strictly correlated with the fibers’ diameter [47], thus the polymer selection and electrospinning parameters. Membranes printed with silver ink showed the greatest conductivity in comparison to those printed with carbon ink, even the volume of the printed ink was 10 times lower on the electrospun membranes.

### 3.3. Cytotoxicity

**Figure 8:**
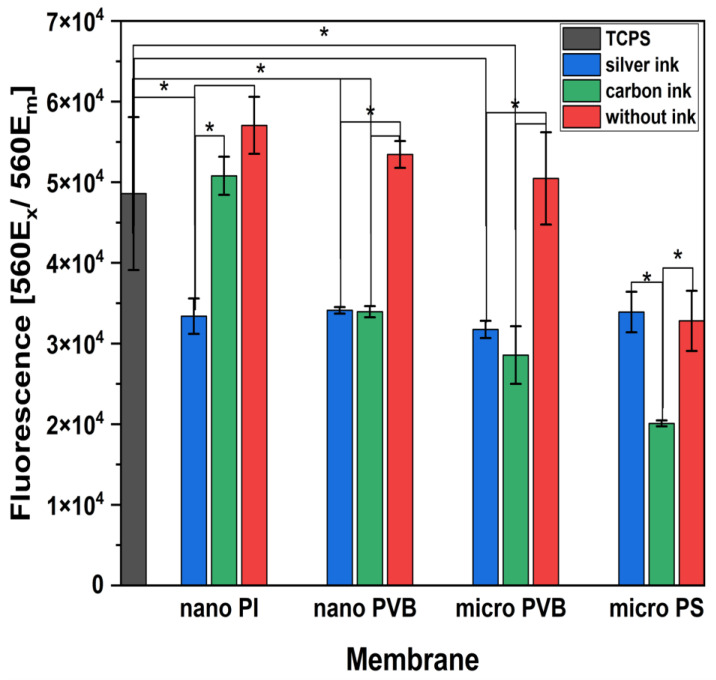
Cytotoxicity test after 24 h of cell culture with the medium incubated for 72 h with PI and PVB nanofibers, PVB and PS microfibers pristine and printed with silver and carbon ink; TCPS was used as a positive control. * Statistical significance calculated with ANOVA, followed by Tukey’s posthoc test, *p* < 0.05; error bars are based on standard deviation. All the micro PS samples showed statistically significant differences with TCPS.

The indirect in vitro cytotoxicity of printed layers was investigated, see Figure 8. Cell culture medium incubated with PI and PVB nanofibers and PVB microfibers and then used for osteoblasts culture did not reduce cell proliferation [65,66]. The level of measured fluorescence for those samples related to the number of living cells was on a similar level as the TCPS-positive control. Even as a different sintering temperature was used for the electrospun membranes printed with silver ink, all of them demonstrated reduced proliferation. Silver nanoparticles are widely applied for antibacterial materials; however, in contact with cells, they could induce a cytotoxic effect [67]. PI membrane printed with carbon ink and sintered at the required temperature (200 °C) did not affect the cells’ development. On the contrary, both PVB membranes printed with carbon ink and sintered at low temperatures reduced cell proliferation. Cells cultured with medium incubated with PS microfibers showed significantly lower proliferation than other tested membranes; moreover, PS has already been reported to possess low biocompatibility [59]. For the sintering temperature of silver and carbon inks not reaching the required value, some additives remained in the membrane. Then they were released to the cell culture medium and the proliferation was reduced.

## 4. Conclusions

In this study, we verified commonly electrospun polymer membranes as substrates for inkjet printing of conductive paths using two standard inks: carbon and silver. All the electrospun membranes were hydrophobic based on nano- and microfibers. We showed that hydrophobic fibers are able to facilitate ink absorption. Electrospun fibers are flexible and elastic but can also be biocompatible and degradable; therefore, they fulfill the typical requirements for substrates in printed electronics and medical sensors. Fibrous membranes printed with silver ink showed lower resistance when compared to carbon ink. Furthermore, the greatest conductivity was obtained for nano PI membrane printed with silver ink, as the sintering temperature could reach the recommended value of 140 °C due to the thermal stability of this polymer. Additionally, the 1 pL droplets of deposited ink resulted in a homogenous layer creating a sufficient conductive path in the electrospun substrate. These results indicated that the most limiting factor for selected nanofiber and microfiber electrospun membranes was the sintering temperature, which should not exceed the T_g_ of polymer used as a substrate. Most importantly, we showed that fiber diameter, hence, pore size, in the membrane had an impact on the printing quality and ink penetration depth. The cytotoxicity studies demonstrated slightly reduced cell proliferation by the electrospun membranes printed with silver and carbon ink, especially for PS. The best biocompatibility results were obtained for the nano PI membrane, which is related again to the sintering temperature of ink required to remove the residues of solvents. This visibility study explored the most important aspects of developing printed electronics on electrospun substrates. We provide guidance in designing smart textiles based on polymer selection, membrane morphology, resistance and biocompatibility.

## Figures and Tables

**Table 1:** Summary of all the electrospinning parameters to produce PVB, PS and PI electrospun membranes.

Electrospun Membrane	Voltage Applied Needle–Collector [kV]	Distance Needle–Collector [cm]	Flow Rate [mL·h^−1^]	Temperature [°C]	Humidity [%]
nano PI	16	15	0.3	22	60
nano PVB	14–15	15	1.0	25	30
micro PVB	10–11	15	1.5	25	30
micro PS	11–12	20	1.5	25	40

**Table 2:** Summary of the printing parameters for silver and carbon ink on the PI and PVB nanofibers, PVB and PS microfibers.

Membrane	Silver Ink		Carbon Ink	
	Voltage [V]	Distance between the Platen and Cartridges [μm]	Voltage [V]	Distance between the Platen and Cartridges [μm]
nano PI	32	1000	40	1000
nano PVB	32	700	40	1000
micro PVB	32	700	40	1000
micro PS	32	1200	40	1200

## Data Availability

The data supporting this article are found within the text.
